# Statistical approaches for differential expression analysis in metatranscriptomics

**DOI:** 10.1093/bioinformatics/btab327

**Published:** 2021-07-12

**Authors:** Yancong Zhang, Kelsey N Thompson, Curtis Huttenhower, Eric A Franzosa

**Affiliations:** Harvard Chan Microbiome in Public Health Center, Harvard T. H. Chan School of Public Health, Boston, MA 02115, USA; Department of Biostatistics, Harvard T. H. Chan School of Public Health, Boston, MA 02115, USA; Infectious Disease and Microbiome Program, Broad Institute of MIT and Harvard, Cambridge, MA 02142, USA; Harvard Chan Microbiome in Public Health Center, Harvard T. H. Chan School of Public Health, Boston, MA 02115, USA; Department of Biostatistics, Harvard T. H. Chan School of Public Health, Boston, MA 02115, USA; Infectious Disease and Microbiome Program, Broad Institute of MIT and Harvard, Cambridge, MA 02142, USA; Harvard Chan Microbiome in Public Health Center, Harvard T. H. Chan School of Public Health, Boston, MA 02115, USA; Department of Biostatistics, Harvard T. H. Chan School of Public Health, Boston, MA 02115, USA; Infectious Disease and Microbiome Program, Broad Institute of MIT and Harvard, Cambridge, MA 02142, USA; Department of Immunology and Infectious Diseases, Harvard T. H. Chan School of Public Health, Boston, MA 02115, USA; Harvard Chan Microbiome in Public Health Center, Harvard T. H. Chan School of Public Health, Boston, MA 02115, USA; Department of Biostatistics, Harvard T. H. Chan School of Public Health, Boston, MA 02115, USA; Infectious Disease and Microbiome Program, Broad Institute of MIT and Harvard, Cambridge, MA 02142, USA

## Abstract

**Motivation:**

Metatranscriptomics (MTX) has become an increasingly practical way to profile the functional activity of microbial communities *in situ*. However, MTX remains underutilized due to experimental and computational limitations. The latter are complicated by non-independent changes in both RNA transcript levels and their underlying genomic DNA copies (as microbes simultaneously change their overall abundance in the population and regulate individual transcripts), genetic plasticity (as whole loci are frequently gained and lost in microbial lineages) and measurement compositionality and zero-inflation. Here, we present a systematic evaluation of and recommendations for differential expression (DE) analysis in MTX.

**Results:**

We designed and assessed six statistical models for DE discovery in MTX that incorporate different combinations of DNA and RNA normalization and assumptions about the underlying changes of gene copies or species abundance within communities. We evaluated these models on multiple simulated and real multi-omic datasets. Models adjusting transcripts relative to their encoding gene copies as a covariate were significantly more accurate in identifying DE from MTX in both simulated and real datasets. Moreover, we show that when paired DNA measurements (metagenomic data) are not available, models normalizing MTX measurements within-species while also adjusting for total-species RNA balance sensitivity, specificity and interpretability of DE detection, as does filtering likely technical zeros. The efficiency and accuracy of these models pave the way for more effective MTX-based DE discovery in microbial communities.

**Availability and implementation:**

The analysis code and synthetic datasets used in this evaluation are available online at http://huttenhower.sph.harvard.edu/mtx2021.

**Supplementary information:**

Supplementary data are available at *Bioinformatics* online.

## 1 Introduction

While DNA shotgun sequencing of microbial communities [i.e. metagenomics, (MGX)] has become a dominant technique to profile community taxonomy and functional potential (Quince *et al.*, 2017), decoding this genetic potential does not directly indicate its transcriptional activity under any given condition. Profiling whole-community RNA, metatranscriptomics (MTX), is an increasingly practical way to link community genetic potential (gene carriage and abundance) to molecular activity (Bashiardes *et al.*, 2016). Given that microbes can vary their transcriptional profiles dramatically among local environmental conditions, MTX is crucial for understanding microbial behavior *in situ*, as demonstrated by a variety of human health applications (Franzosa *et al.*, 2014; Heintz-Buschart *et al.*, 2016; McNulty *et al.*, 2011; Schirmer *et al.*, 2018) and studies of the environment (Baldrian *et al.*, 2012; Coolen and Orsi, 2015; Frias-Lopez *et al.*, 2008; Poretsky *et al.*, 2009; Salazar *et al.*, 2019; Vorobev *et al.*, 2020). However, since validated computational and statistical workflows for MTX analysis have been few in number (Franzosa *et al.*, 2015; Giannoukos *et al.*, 2012), many MTX analyses have continued to rely upon techniques borrowed from single-organism RNA-seq (Dillies *et al.*, 2013; Lin *et al.*, 2016), despite gaps in their modeling assumptions and lack of validation for microbial communities.

**Fig. 1.**
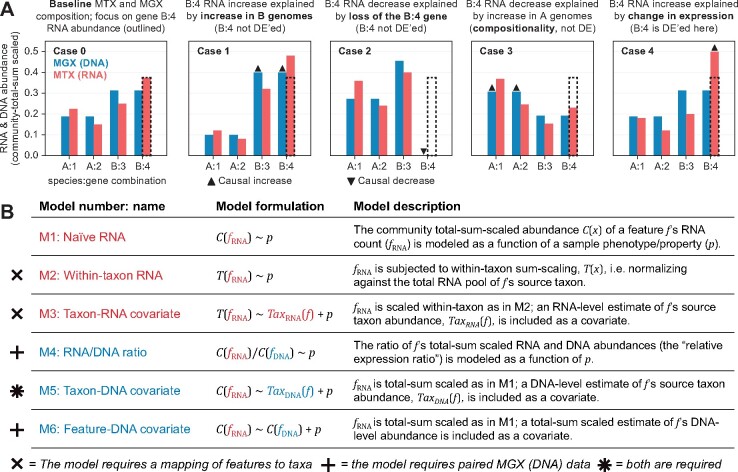
Normalization and DE models for microbial community transcript abundances. (**A**) Each panel corresponds to a simple conceptual community (two species, A and B, each contributing two genes) assayed by MTX and MGX sequencing. Case 0 represents a reference condition, while Cases 1–4 correspond to perturbations of the reference. While the RNA abundance of gene B:4 (dashed outline) differs under each perturbation, only in Case 4 is the change attributable to DE rather than gene copy-number variation. (**B**) A summary of six linear models for assaying DE of a MTX feature *f* with respect to a sample phenotype/property *p*. Models 2–6 incorporate transformations and covariates aimed at minimizing spurious DE signals from gene copy number

A variety of factors complicate differential expression (DE) analysis from MTX data, some of which are shared with single-organism RNA-seq. For example, MTX transcript abundances are initially measured as integer read counts, which are sensitive to sequencing depth and transcript length and must be modeled appropriately. These counts are often inflated with zero values incorporating technical non-detection events and biological variability (Mallick *et al.*, 2017). Such properties are accounted for by single-organism RNA-seq methods (Love *et al.*, 2014; Robinson *et al.*, 2010; Trapnell *et al.*, 2013) and workflows that adapt those methods to microbial communities (Kim *et al.*, 2016; Martinez *et al.*, 2016; Ni *et al.*, 2016; Westreich *et al.*, 2018). However, MTX data differ from single-organism RNA-seq in a critical way: the RNA abundance of a gene in a community tends to vary strongly with the gene’s underlying copy number (DNA abundance; Fig. 1A). While this phenomenon can assert itself via copy number or ploidy changes in single organisms as well (Dillies *et al.*, 2013; Lin *et al.*, 2016), such events are typically rare. Conversely, abundances of microbial community members (and hence their encoded genes) routinely vary by orders of magnitude across related environments. This variation complicates DE analysis in MTX data by inducing changes in transcript abundance that are independent of differential regulation, in addition to exaggerating the effects of compositionality and the dynamic range of MTX measurements. Pervasive genomic plasticity and mobility within communities can additionally alter gene copy number across communities (Quince *et al.*, 2017), leading to non-regulatory changes in transcript abundances.

To account for this baseline dependency between community functional activity (MTX) and potential (MGX), procedures are needed to estimate the ‘relative expression’ of a function, i.e. the extent to which it is over- or under-expressed relative to the number of community genes that encode it. For example, (Klingenberg and Meinicke, 2017) describe a ‘taxon-specific scaling’ normalization approach within MTX species/taxon. This approach, which approximately transforms a MTX dataset into an aggregate of single-organism RNA-seq datasets, assumes that all genes within a species have constant coding potential. Taxon-specific scaling loses power when community genes cannot be assigned to a reference species or taxonomic bin, which can range from a few percent to >90% among community types (Quince *et al.*, 2017). Alternatively, when samples are profiled with paired MTX and MGX sequencing, normalizing each community gene’s RNA abundance by its DNA abundance is a more direct approach (Lloyd-Price *et al.*, 2019; Salazar *et al.*, 2019): one which accounts for gene copy number variation within species via per-gene DNA abundance estimates while simultaneously applying to genes of unknown taxonomy. However, this of course requires MTX and MGX profiles to be generated from the same samples, and systematic evaluation of the effectiveness of these procedures for DE in communities has not yet been sufficiently explored.

To address these gaps, we present a systematic effort to develop and evaluate statistical approaches for community DE analysis from MTX data. We tested six base linear models for identifying DE that make different assumptions about MTX normalization, MGX availability and the relationship between DNA and RNA copy numbers (Fig. 1B). We additionally explored modeling choices for management of zero-inflation in MTX data, including pre-filtering likely technical zeros and modeling feature presence/absence via logistic regression. Combinations of normalization options, pre-filters and tuning parameters were applied to a battery of synthetic MTX and MGX datasets incorporating spiked DE and confounding biological/technical signals, thus enabling rigorous performance assessment. These results allowed us to make specific best-practice recommendations for MTX analysis given the presence/absence of paired MGX data. We close by demonstrating the performance of these methods in a case study of human gut microbiome gene expression during inflammatory bowel disease (IBD).

## 2 Materials and methods

We systematically evaluated a series of statistical approaches for DE analysis in MTX data using a combination of synthetic and real-world sequencing data. This section (i) summarizes our procedures for generating synthetic MTX and MGX data with spiked phenotype associations; (ii) defines six MTX normalization models, three pre-filtering procedures and additional tuning parameters underlying our statistical approaches and (iii) describes how these approaches were applied to synthetic and real-world data.

### 2.1 Simulating paired metatranscriptomes and metagenomes

We simulated microbial communities based on the 100 most-prevalent species from the healthy human gut microbiome (Lloyd-Price *et al.*, 2017) which we then used as templates for generating synthetic MTX and MGX sequencing count data with spiked phenotypic associations (including simulated DE). The details of this process are summarized here and expanded in Supplementary Note S1. To simulate microbial community compositions, we sampled species in proportion to their empirical prevalence and selected abundances from fitted log-normal distributions. We modeled species pangenomes as bags of 1K genes drawn from a larger pool of 2K gene families. A community strain carriage of a given species was randomly drawn from the species’ 1K genes (gene content thus varies across simulated strains of a species, modeling genomic plasticity). Each gene family was additionally associated with a reference (log-mean) expression level *m* drawn from *N*(0, 1) with per-sample expression values drawn from log *N*(*m*, 1).

Samples were randomly assigned to a binary phenotype. One or more phenomena (read depth, species abundance, gene presence/absence and gene expression level) were associated with this phenotype by shuffling their values to induce rank-correlations of a pre-specified strength. Gene-phenotype associations involving expression level are positive for DE in subsequent model evaluations. We simulated 11 datasets of *N *=* *100 samples containing different combinations of phenomena spiked to associate with sample phenotype (Table 1 and Supplementary Table S1). For example, the ‘null-bug’ dataset contains species-phenotype associations but no positive DE relationships, while the ‘true-combo-dep-exp’ dataset contains a mix of positive DE relationships and a potentially confounding sequencing depth association.

**Table 1.** Properties of 11 synthetic datasets (columns) used in community DE benchmarking

	null	null- bug	null- enc	null- dep	true- exp	true-exp- med	true-exp- low	true-combo- bug-exp	true-combo- dep-exp	group-null- enc	group- true-exp
**% of transcripts associated (DE’ed) with phenotype**	––	––	––	––	**10%**	**10%**	**10%**	**10%**	**10%**	––	**10%**
**Strength of DE relationships**	––	––	––	––	**High**	**Med**	**Low**	**High**	**High**	––	**High**
**% of taxa strongly confounded with phenotype**	––	**10%**	––	––	––	––	––	**50%**	––	––	––
**% of gene gain/loss strongly confounded with phenotype**	––	––	**10%**	––	––	––	––	––	––	**10%**	––
**Sequencing depth confounded with phenotype**	––	––	––	**Yes**	––	––	––	––	**Yes**	––	––
**Gene taxonomy is unknown; trends at orthogroup level**	––	––	––	––	––	––	––	––	––	**Yes**	**Yes**
	**←11 synthetic MTX + MGX datasets →**										

*Notes*: Datasets of *N* = 100 paired MTX and MGX samples based on healthy human gut microbiomes were spiked to include associations between a synthetic case: control phenotype and some combination of sequencing depth, microbial species, gene presence/absence and gene expression. Expression-phenotype associations are DE positives, while other associations may introduce spurious (FP) signals of DE. ‘––’ implies 0% or ‘n/a.’ These datasets are described further in Supplementary Table S1.

To simulate MGX count data, reads were sampled up to a sample’s sequencing depth and assigned to sample genes in proportion to the abundance of the gene’s source species. MTX count data were simulated similarly, with reads assigned to transcripts (genes) in proportion to the abundance of their source species and per-sample gene expression value.

### 2.2 Analytical approaches for DE analysis in metatranscriptomic data

We evaluated a series of linear models for community DE analysis incorporating one of six gene-copy normalization methods and three upstream zero-filtration methods alongside other parameter choices. Models were implemented using the glm function of R’s ‘stat’ package. Unless otherwise stated, species, gene and transcript counts were initially total-sum-scaled (to adjust for sample read depth) and then log-transformed (for variance-stabilization). Zero values surviving filtration were additively smoothed by half the corresponding feature’s smallest non-zero measurement prior to log-transformation.

#### 2.2.1 Approaches for gene-copy number normalization

We considered six approaches for normalizing MTX abundances during DE analysis (M1-6, summarized in Fig. 1B). M1 (the naïve baseline) incorporates only community-level total-sum-scaling over MTX features (no accounting for gene-copy variation). M2 and M3 improve on M1 by incorporating within-taxon total-sum-scaling, in which a feature’s per-sample RNA abundance is divided by the total RNA abundance contributed by its source species. This procedure was described as ‘taxon-specific scaling’ in (Klingenberg and Meinicke, 2017) and, where feature-taxon relationships are known, can compensate for variation in RNA abundance driven by changes in underlying species abundance. M3 refines this approach by adding the species’ total RNA contributions as a model covariate. This value serves as a proxy for taxon abundance in the absence of MGX data, with taxon (genomic) abundance providing an estimate of per-feature gene copy number (assuming constant encoding).

Three additional normalization procedures (M4–M6; Fig. 1B) are enabled by the availability of paired MGX sequencing data. M5 is an analog of M3 based on community-scaled MTX features but incorporating a more accurate DNA-level estimate of taxon abundance. M5 (like M2 and M3) can compensate for MTX variation driven by source species abundance but not gene-copy variation *within* species, e.g. gene duplication or loss. Two final approaches (M4 and M6) directly relate the expression of a gene with its underlying DNA copy number. M4 models the ratio of a feature’s MTX (RNA) abundance to its MGX (DNA) abundance as a measure of ‘relative expression’ (i.e. transcripts detected per gene copy). These ratios are computed on smoothed, community-normalized MTX and MGX measurements and then log-transformed prior to model evaluation. M6 normalizes MTX feature expression for gene copy number by including the feature’s MGX abundance as a model covariate, thus emphasizing the feature’s ‘residual expression’ in DE analysis. Notably, M4 and M6 do not require knowledge of gene-taxon relationships, making them amenable to analyses of unclassified MTX features or community totals (i.e. sums over known and unclassified contributions).

#### 2.2.2 Pre-filtering technical zeros and other tuning parameters

In addition to the six models of MTX normalization introduced above, we considered three approaches for balancing biological and technical zeros in MTX data: ‘lenient’ filtering, which excludes features that are *always* zero-valued in MTX or gene-copy estimates (e.g. MGX), ‘semi-strict’ filtering, which filters samples per-feature if *both* their MTX and gene-copy values are zero and ‘strict’ filtering, which filters samples if *either* their MTX or gene-copy value are zero (Supplementary Fig. S1). When a feature was removed prior to DE analysis due to filtering, it was counted as ‘negative for DE’ for the purposes of performance evaluation. As an alternative approach to handling zero values, we evaluated models for DE analysis linking transcript presence/absence with sample covariates using logistic regression, while optionally incorporating MTX read depth as an additional covariate. The total space of MTX analyses benchmarked in this study (gene-copy normalization model × pre-filtering approach × linear versus logistic regression × optional sequencing depth covariate) is enumerated in Supplementary Table S2.

#### 2.2.3 Performance evaluation

We evaluated the sensitivity and specificity of community DE analysis methods across synthetic datasets. A positive DE signal was defined as a transcript that was purposefully spiked to associate with sample phenotype; all other transcripts are defined to be negative for DE. A true positive (TP) was conservatively defined as a feature whose phenotype model coefficient was significantly different from 0 after FDR correction, i.e. having Benjamini–Hochberg FDR *q *<* *0.05 (Benjamini and Hochberg, 1995). A feature that was negative for DE but had a nominally significant association with phenotype (*P *<* *0.05) was classified as a false positive (FP). We report the TP rate (TPR) as the fraction of positives that were TPs and the FP rate (FPR) as the fraction of negatives that were FPs under the above definitions. An ideal model will have FPR of 0.05 (the theoretical type-I error rate) on all datasets and a TPR of 1.00 on datasets containing DE positives (TPR is not defined for datasets lacking spiked expression associations; Table 1).

### 2.3 Analysis of differentially expressed pilins in IBD

We used MTX and MGX data from the IBD Multi-omics Database (IBDMDB) cohort (Lloyd-Price *et al.*, 2019) to evaluate the real-world performance of a subset of community DE methods. Data were downloaded from https://www.ibdmdb.org/ in December 2020. We focused on 800 samples with paired MTX and MGX profiles from 109 longitudinally-sampled participants: 52 with Crohn’s disease (CD), 30 with ulcerative colitis (UC) and 27 non-IBD controls. Longitudinal CD and UC samples from this cohort had been previously defined as ‘dysbiotic’ if they deviated from the ‘cloud’ of healthy microbiome configurations represented among non-IBD MGX profiles (with ‘deviation’ defined as a median Bray–Curtis distance to non-IBD samples exceeding the 90th percentile of non-IBD comparisons) (Lloyd-Price *et al.*, 2019). IBDMDB had been previously analyzed using MetaWIBELE v0.3.8 (https://huttenhower.sph.harvard.edu/metawibele/): a method that (i) assigns candidate functions and taxonomy to genes in a microbial gene catalog and (ii) prioritizes those functions on the basis of functional, ecological and phenotypic properties. Based on this information, we selected a subset of 113 proteins for DE analysis that were (i) enriched in MGX samples from dysbiotic time points, (ii) taxonomically annotated to *Escherichia coli* and (iii) functionally annotated as pilin-related proteins on the basis of Pfam domain assignments (release Pfam32) (Finn *et al.*, 2016).

MGX and MTX abundance profiles containing pilin-related proteins were initially community total-sum-scaled and then filtered using the semi-strict method to balance removal of technical zeros and loss of dysbiotic samples. Non-filtered RNA and DNA values were then log-transformed and smoothed following the procedures outlined above for synthetic data. Each pilin feature was analyzed for DE under the ‘dysbiosis’ phenotype using three gene-copy normalization methods (M3, M4 and M6; Fig. 1B). We re-implemented these methods in the lmerTest R package (Kuznetsova *et al.*, 2017) to accommodate subjects’ repeated measures as a random effect, ‘(1|subject).’ ‘Diagnosis’ (CD versus UC versus Non-IBD), ‘antibiotic’ use and consent ‘age’ were added as additional covariates relevant to the IBDMBD cohort. Nominal *P*-values for UC- and CD-specific pilin DE in dysbiosis were subjected to multiple hypothesis testing correction using the Benjamini–Hochberg method with a FDR threshold of 0.25.

## 3 Results

We evaluated approaches for detecting DE from metatranscriptomic (MTX) sequencing with an emphasis on six methods for normalizing MTX data to account for gene-copy variation (Fig. 1). We separately evaluated (i) the impact of pre-filtering MTX and MGX data for likely technical zeros, (ii) model performance on communities of unclassified taxonomy and (iii) additional tuning parameters. Evaluations were performed on synthetic MTX and MGX datasets spiked to contain true DE signals as well as biological/technical confounders (Table 1 and Supplementary Table S1).

### 3.1 Statistical models accounting for underlying variation in gene copy number facilitate DE discovery in MTX

We initially evaluated the performance of six approaches to MTX normalization (M1-6, defined in Fig. 1B) using ‘strict’ pre-filtering of MTX zeros (Section 2). For models M3-6 (i.e. those incorporating an estimate of gene copy number), strict per-filtering excludes samples from the analysis of a given feature if the feature’s per-sample RNA abundance or gene-copy estimate were zero. The impact of choosing a less-strict pre-filtering approach is explored in the next section.

**Fig. 2.**
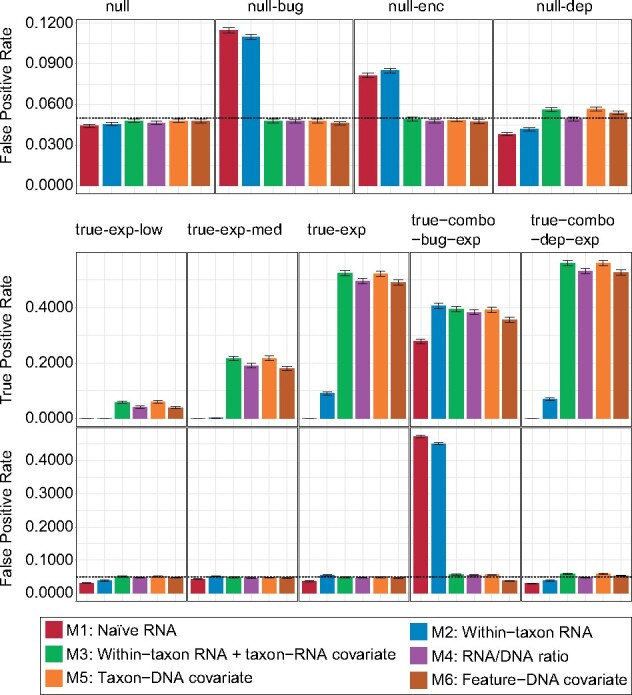
DE models for MTX accounting for underlying variation in gene copy number control FP rates while maintaining statistical power. We evaluated the performance of six models for DE in MTX data (M1-6; Fig. 1B) on nine synthetic datasets with paired MTX and MGX measurements of known taxonomy (Table 1). ‘Null’ datasets (top row) contained no positive DE signatures and were evaluated in comparison with the theoretical nominal type-1 error rate only (dashed lines; FPR = 0.05). ‘True’ datasets contained 10% positive DE relationships and were evaluated on the basis of their sensitivity (accounting for multiple hypothesis correction; middle row) versus nominal type-I error rate (bottom row). Error bars reflect the 95% CI for percentages

All models (M1-6) displayed close to optimal specificity (FPR ∼ 0.05) on the ‘null’ dataset, which contained no spiked phenotype associations (Fig. 2). As expected, model M1 (which incorporates only total community-level MTX normalization) was prone to FP DE detection when RNA counts varied due to confounding changes in gene copy number. This was true for confounding variation in species abundance (the ‘null-bug’ dataset; FPR = 0.110) and gene presence/absence (the ‘null-enc’ dataset; FPR = 0.082). The inability to isolate signals of gene expression from gene-copy variation further limited model M1’s ability to detect even strong positive DE signals (TPR = 0). Surprisingly, the next-most sophisticated model (M2, incorporating within-taxon normalization), suffered from similarly high FP rates in the presence of confounding DNA-level species and gene variation (FPR = 0.110 and 0.085), possibly due to the amplification of noise within species at low absolute RNA abundance. M2 was somewhat more successful than M1 at detecting strong DE signals (TPR = 0.091).

In contrast, the four models incorporating estimates of gene copy number (M3-6) continued to achieve near-optimal specificity even in the presence of confounding signals (Fig. 2). Models M3 and M5, which use taxon-level RNA and DNA (respectively) to estimate copy number, benefit here from strict pre-filtering of RNA zeros (e.g. by avoiding the potential to confuse RNA zeros resulting from gene loss with DE in the ‘null-enc’ dataset). M3 and M5 displayed slightly elevated FPs when sequencing depth was confounded with sample phenotype (‘null-dep’ dataset; FPR = 0.057 for M3 and 0.056 for M5), as did model M6, which uses a per-feature DNA covariate to adjust for gene copy number (FPR = 0.054). Model M4, which is based on the RNA/DNA ratio, was robust to FPs from sequencing depth confounding (FPR = 0.0493) but more likely than M6 to produce a FP given extensive confounding with community structure (‘true-combo-bug-exp’ dataset, FPR = 0.0557 for M4 versus 0.0382 for M6).

Methods M3-6 were broadly similar with respect to sensitivity for positive DE relationships (Fig. 2). Indeed, the strength of DE (from weak to strong across the ‘true-exp-low,’ ‘true-exp-med’ and ‘true-exp’ datasets) was a much stronger determinant of sensitivity than choice of model among M3-6. That said, we did observe a trend for models M3 and M5 to be slightly more sensitive than models M4 and M6 (e.g. TPR of 0.218 and 0.217 versus 0.191 and 0.181 among medium-strength DE trends), possibly due to the greater influence of read-level noise on the per-feature gene-copy estimates used by models M4 and M6. In summary, when using strict pre-filtering of RNA zeros, within-taxon RNA scaling with a taxon-RNA covariate (model M3) provided a good balance of sensitivity and specificity for DE in MTX in the absence of paired MGX data (but requires knowledge of gene-taxon relationships). If paired MGX data are available, models M4 and M6 perform well and do not require knowledge of gene-taxon relationships.

### 3.2 Prefiltering zero values distinguishes low-expression genes from non-encoding or non-detection events

In MTX, transcript measurements (based on integer read counts) are commonly zero-inflated to an even greater extent than single-organism RNA-seq. This is due to the high ratio of MTX features (∼0.1–1 M transcripts) to sequencing depth (∼10–100 M reads) in most communities and the striking dynamic range among those features (6–8 orders of magnitude) (Lloyd-Price *et al.*, 2017, 2019). Many MTX zeros are due to non-detection events (expression below the limit of detection of a given sequencing depth) or non-encoding events (gene loss within a community’s taxon lineage), instead of only absence/down-regulation of expression.

To assess the impact of MTX zeros on DE analysis, we tested three zero pre-filtering strategies across the six statistical models introduced above (Supplementary Fig. S1). The first strategy, ‘lenient filtering’, treats a feature as uninformative for DE analysis if it was always zero at the RNA level (all models) or always zero in gene-copy estimates (models M3-6), but otherwise considers all sample values. The second strategy, ‘semi-strict filtering’, excludes a sample from the analysis of a given feature under models M3-6 if the feature’s RNA count and gene-copy estimate are both 0, but includes the sample if one or both values are non-zero. The final strategy, ‘strict filtering’ (as applied in Fig. 2), excludes a sample if *either* the RNA or gene-copy estimate is zero, thus including only those samples where both values are non-zero (conservatively treating all MTX zeros as probable non-detection events).

**Fig. 3.**
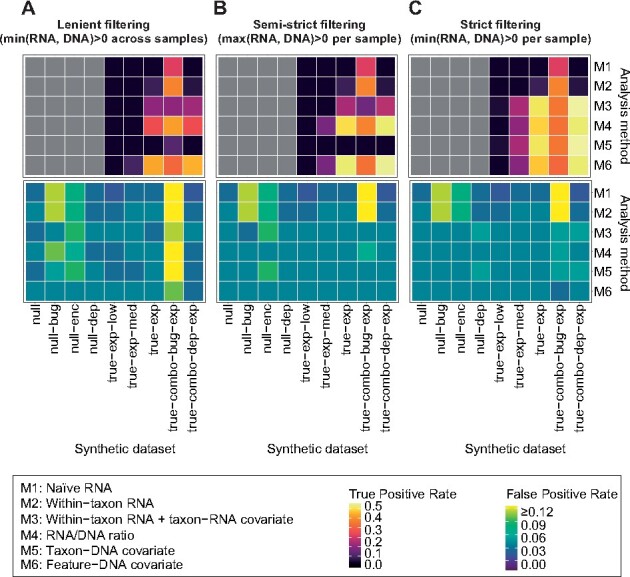
DE models for MTX benefit from pre-filtering of probable technical zeros. We assessed three pre-filtering strategies for balancing biological and technical zeros in MTX data. (**A**) Under ‘lenient’ pre-filtering, features were analyzed for DE if they were ever detected (non-zero) at the RNA level (or gene-copy level, where applicable). (**B**) Under ‘semi-strict’ pre-filtering, samples were excluded if both a feature’s RNA count and gene-copy estimate (where applicable) were zero. (**C**) Under ‘strict’ pre-filtering, samples were excluded if either a feature’s RNA count or gene-copy estimate were zero. Features that were excluded from analysis were scored as ‘not DE’ (i.e. negatives). Gray cells indicate ‘undefined’ TPR for datasets lacking positive DE signals

Overall, pre-filtering zeros that cannot contribute to significant transcriptional differences improved the performance of statistical models for DE discovery (Fig. 3). This is particularly intuitive for ‘uninformative’ zeros, i.e. samples with no DNA or RNA for a given gene resulting from non-encoding or non-detection events, which can inflate both FPs and false negatives (FNs). Lenient pre-filtering resulted in the greatest expansion in FPs across models, especially as applied to datasets with confounding species abundance and gene presence/absence signals (Fig. 3A and Supplementary Fig. S2); specificity was improved under semi-strict filtering (Fig. 3B and Supplementary Fig. S3) and maximized under strict filtering (Fig. 3C). Sensitivity followed a similar increasing trend, with a notable jump for the models using taxon-level gene-copy estimates (M3 and M5) under strict versus semi-strict filtering. M3 and M5 with semi-strict filtering were additionally prone to FPs in the presence of confounding genomic plasticity (the ‘null-enc’ dataset), as their gene-copy estimates are not informative for RNA zeros resulting from low expression versus gene loss. Notably, the model adjusting RNA for DNA copy number as a covariate (M6) most consistently controlled FPs while maintaining statistical power across the three pre-filtering approaches. Conversely, the taxon-RNA covariate model (M3), i.e. the best-performing approach in the absence of MGX data, was only reliable under strict pre-filtering of zero values.

### 3.3 Accounting for underlying differences in metagenomic abundance enables DE detection in the absence of taxonomic annotation

An important component of many microbial communities is the pool of DNA or RNA features that cannot be confidently assigned to specific taxa via homology or *de novo* assembly and binning. While the analyses above presumed that all features could be assigned to specific taxa (a prerequisite for models M2, M3 and M5; Fig. 1B), this assumption will be violated for a fraction of genes in most communities and for potential majorities of genes in poorly characterized communities. To address this phenomenon in the context of community DE analysis, we performed additional evaluations of models M1, M4 and M6 on two synthetic MTX datasets lacking taxonomic provenance (Table 1 and Supplementary Table S1). There, positive DE and confounding signals were spiked consistently over gene orthogroups as they occurred across community species (modeling scenarios where all species contributing a certain function respond in the same way to a given condition). We then performed DE modeling on summed orthogroup abundances.

**Fig. 4.**
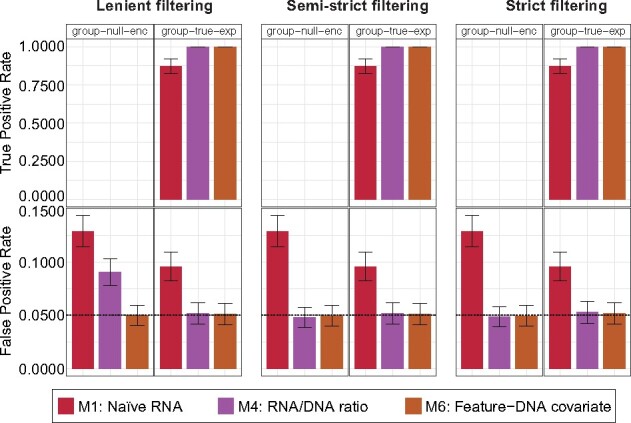
Adjusting RNA for DNA gene copy number provides consistently higher performance in DE analysis for communities containing unknown taxonomy. We assessed the performance of community DE models that do not require a mapping of MTX features to taxa (M1, M4 and M6) on communities of unknown taxonomy. One of these community datasets (‘group-null-enc’) was spiked with confounding gene presence/absence signals, while a second (‘group-true-exp’) contained positive DE signals. Spikes were generated at the gene family (orthogroup) level. Each method was evaluated in combination with three pre-filtering schemes for managing zero inflation (Supplementary Fig. S1). Error bars reflect the 95% CI for percentages

In the presence of confounding between gene presence/absence and phenotype (the ‘group-null-enc’ dataset), the naïve model (M1) was particularly vulnerable to FPs (FPR = 0.129; Fig. 4). Models M4 (RNA/DNA ratio) and M6 (feature-DNA covariate) achieved the target specificity of FPR ∼ 0.05 for these data under semi-strict or strict filtering of zero values, but M4 experienced elevated FPR with lenient filtering (FPR = 0.091). When applied to positive DE signals (the ‘group-true-exp’ dataset), models M4 and M6 were both highly sensitive and optimally specific independent of pre-filtering. In contrast to within-species DE (Fig. 2), model M1 was more effective at recovering positive community-level DE (TPR = 0.87), but continued to report FPs even in the absence of specific confounding signals (FPR = 0.096). These results stress that modeling adjusted RNA values to account for underlying variation in gene copy number consistently provides lower FPR and effectively aids in DE discovery in microbial communities containing unknown taxonomy.

### 3.4 Gene-copy normalization improves transcript presence/absence modeling

We separately evaluated the performance of six MTX normalization models (Fig. 1) in a logistic regression framework with an optional sequencing depth covariate. This approach directly models transcript presence/absence—rather than abundance, as above—as a function of sample covariates (including the phenotype of interest). We reasoned that this approach might be effective in detecting DE relationships at the level of transcript detection/non-detection, which proves to be more biologically relevant feature, while more elegantly handling genomic plasticity (gene gain/loss) and zero inflation.

Modeling trends incorporating logistic regression were broadly similar to those achieved using linear regression (Supplementary Fig. S4). Specifically, models incorporating gene copy-number normalization (M3-6) outperformed unadjusted within-community (M1) and within-taxon (M2) MTX normalization. Sensitivity was globally weaker after reducing continuous MTX abundance values to presence/absence calls; thus, we consider the logistic regression approach to be a supplement (but not alternative) to the previously-benchmarked linear regression models. The feature-DNA covariate model (M6) proved to be particularly specific in this domain, while models M3 and M5 (which use taxon-level estimates of gene copy number) were less robust to confounding gene presence/absence signals. Surprisingly, the inclusion of a sequencing depth covariate did little to improve specificity and tended to reduce power (sensitivity) across models, possibly due to the much lower degree to which it influences presence/absence than abundance for most features.

### 3.5 Adjusting RNA with DNA copy number as a covariate effectively identifies DE’ed inflammation-associated *Enterobacteriaceae* pilins in the human gut

Finally, we compared the behavior of selected well-performing models from the synthetic evaluations (M3, M4 and M6) on 800 stool samples with paired MTX and MGX data from the IBDMDB cohort (Lloyd-Price *et al.*, 2019). These samples longitudinally span 109 subjects with three primary phenotypes (27 non-IBD controls, 52 Crohn’s disease or CD and 30 ulcerative colitis or UC). A subset of samples from CD/UC subjects were previously defined (Lloyd-Price *et al.*, 2019) as ‘dysbiotic’ if they deviated from the cloud of healthy microbiome configurations covered by controls (Section 2). When evaluating genes from this dataset for potential community DE, we filtered samples using the semi-strict approach to maximize the number of dysbiotic time points available for analysis. Based on our findings above, M6 was expected to be more robust to this choice than M3 or M4 (Fig. 3).

We focused our analysis on 113 pilin-family proteins contributed by *E.coli* with potential DE in the dysbiosis state. *E.coli* is frequently implicated in IBD pathogenesis (Darfeuille-Michaud, 2002; Rolhion and Darfeuille-Michaud, 2007) where its pilin-family proteins play roles in bacterial adhesion, co-aggregation and biofilm formation (Hasegawa *et al.*, 2009; Park *et al.*, 2005). The pilin-family proteins selected here had been previously prioritized on the basis of their increased MGX abundance in dysbiotic samples (Section 2), and we hypothesized that they might exhibit additional increases in functional activity during inflammation (e.g. if they were playing roles as ‘drivers’ of dysbiosis versus ‘passengers’ in more-abundant *E.coli* genomes).

**Fig. 5.**
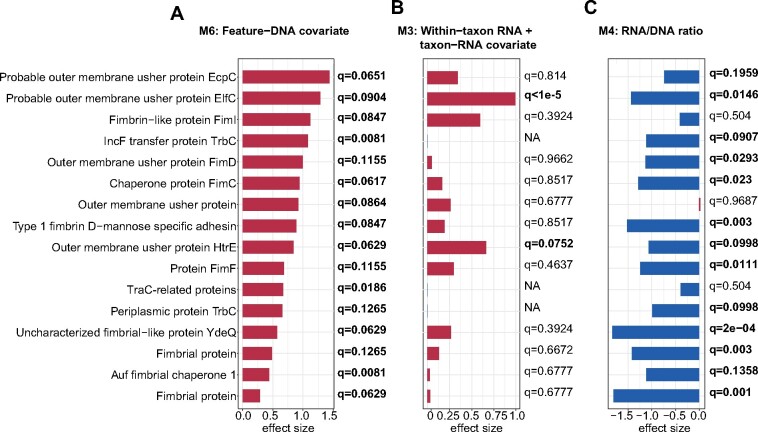
DE of *E.coli* pilin-like proteins in the IBD gut microbiome. We previously prioritized 113 pilin-family proteins assigned to *E.coli* for roles in IBD-associated inflammation based on their MGX properties. We surveyed these proteins for differential functional activity during inflammation using three models of community DE that performed well during synthetic evaluations (Fig. 2). (**A**) The feature-DNA covariate model (M6) identified 16 genes with significantly elevated expression among dysbiotic samples (FDR *q *<* *0.25 as emphasized in bold text). (**B**) The taxon-RNA covariate model (M3) tended to agree with these trends in sign but not statistical significance, while (**C**) the RNA/DNA ratio model (M4) tended to identify these trends as significant in the opposite direction. M3- and M4-specific trends are further compared in Supplementary Figure S5

DE analysis of IBDMDB data using the feature-DNA covariate model (M6) highlighted a specific and dramatic transcriptional increase in *E.coli* genes encoding pilin-related proteins in dysbiotic samples, thus supporting their potential active role in IBD-associated inflammation (mixed-effects linear model, FDR *q *<* *0.25; Fig. 5A). Since ground-truth DE status is not known for these features, we focused our evaluation on comparisons between results returned by models that had performed well on synthetic data (M3, M4 and M6). Among the 16 significantly up-regulated pilin-related proteins predicted by M6, the taxon-RNA covariate model (M3) tended to agree that these proteins were up-regulated, but only two achieved statistical significance (Fig. 5B). M3 additionally called 11 pilins as significantly up-regulated in dysbiosis and 3 as significantly down-regulated (Supplementary Fig. S5). While these discrepancies may indicate unique biological signals detected by M3, it is also possible that they arise from violations of model M3’s assumption of constant gene copy number, which is particularly inappropriate for highly strain-variant clades like *E.coli*.

Surprisingly, while the RNA/DNA ratio model (M4) tended to agree with M6 in its assessments of the statistical significance of pilin DE (Fig. 5C), it did so projecting trends with opposite (negative) sign for most pilins (Supplementary Fig. S5). This behavior is counter to biological intuition, in which inflammation-associated *E.coli* would be more likely to upregulate pilus structures. Where *E.coli’*s MGX abundance is also expanded in dysbiotic samples, RNA/DNA ratios for *E.coli* genes may be depressed to an excessive degree, leading to spurious evidence of down-regulation. Indeed, such interactions between species abundance and small RNA values (allowed under semi-strict filtering) were observed for M4 in the synthetic evaluations (Fig. 3B), where they provided an uncommon point of differentiation between models M4 and M6 (with M6 proving more robust to small values generally, consistent with its behavior in this application).

## 4 Discussion

Shotgun DNA sequencing of microbial communities (MGX) is a highly utilized technology for profiling microbial community composition and functional potential, and shotgun RNA MTX continues to join this approach as a powerful tool for probing community functional activity. However, both due to experimental challenges and computational limitations, MTX remains underutilized. To partially address these challenges, we performed a rigorous evaluation of candidate statistical approaches for detecting community DE of microbial genes and gene families from MTX data.

A defining challenge of statistical analysis of MTX data is the dependence of a gene’s RNA count on its underlying MGX copy number. Failure to account for this coupling leads to extensive spurious community DE signals in the presence of confounding variation in taxon abundance or genomic plasticity, and it can also obscure true DE. We strongly recommend employing DE models in MTX that adjust for gene-copy number with either a taxon-level RNA estimate (M3) or, when paired MGX data are available, a direct feature-level DNA measurement (M6). We further emphasize that, while gene-copy normalization is possible in the absence of paired MGX data, it is limited to genes of known taxonomy. Hence, generating and analyzing paired MGX data for MTX samples is recommended where feasible.

Statistical analysis in MTX is additionally complicated by zero-inflation, which is driven by the interaction between MTX features’ vast numbers, their dynamic range and detection limits imposed by sequencing depth. We explored combining gene-copy normalization with pre-filtering strategies to reduce the impact of technical zeros on detecting DE from MTX data. Strict filtering of MTX zeros was particularly important for maximizing specificity and, more surprisingly, sensitivity in models using taxon-level estimates of gene copy number (including M3). While strict filtering results in greater sample loss (reduced power) for M3, this effect was outcompeted by improved signal-to-noise ratio from removed technical zeros. In contrast, semi-strict filtering under M3 was prone to mistaking RNA non-detection for reduced expression when species abundance was low (leading to FNs), in addition to mistaking gene loss for reduced expression at any species abundance (leading to FPs). Notably, the feature-DNA covariate model (M6) was largely robust to pre-filtering strategy, including in data lacking taxonomic provenance. Coupled with the interpretability of M6’s ‘residual expression’ values, and the potential instability of the RNA/DNA ratio (model M4) in the presence of small values, we highlight M6 as our current preferred model of community DE when paired MGX data are available.

We focused in this work on DE methods for MTX that could be conveniently implemented in existing linear modeling frameworks while simultaneously compensating for the challenging properties of MTX data. Adapting such frameworks is useful given their support for complex covariate structures, which are critical in (e.g.) epidemiological studies of the human microbiome. However, it remains possible that statistical methods developed *de novo* for DE in MTX might perform better than our best performers, and more research in this area is warranted. Relatedly, there are a number of potential avenues for modifying the synthetic data generation framework we used to benchmark DE methods that could be applied in future evaluations. Our framework simulated microbial communities based on empirical species prevalence and abundance distributions, but assumed (i) a high, constant rate of gene encoding within strains of a species (80%) and (ii) random log-normal gene expression within and across species. Alternatively, gene-level properties could also be modeled on empirical distributions, and doing so might resolve observed differences in model performance on synthetic versus real-world data (for example, by better representing the extensive genomic plasticity of species like *E.coli*). In addition, more accurately modeling differences in species’ global transcriptional activities would impact the accuracy of M3’s RNA-based estimates of taxon abundance (which may be overly optimistic in our evaluation).

Notwithstanding these options for future work, the simulation and evaluation framework presented here provides a strong foundation for microbial community DE analysis from MTX data, providing models that accurately account for normalization, zero-inflation and confounding of microbial growth and genomic plasticity with transcriptional changes linked to environmental parameters or phenotypes.

## Supplementary Material

btab327_Supplementary_Data
