# Supplementary material for: Statistical approaches for differential expression analysis in metatranscriptomics
Source: Bioinformatics. 2021 Jul 12;37(Suppl 1):i34–41. doi: 10.1093/bioinformatics/btab327 (PMC8275336; doi:10.1093/bioinformatics/btab327)
Supplement: btab327_Supplementary_Data [file btab327_supplementary_data.pdf]

Supplementary Information for

Statistical approaches for differential expression analysis in metatranscriptomics

Yancong Zhang[1,2,3], Kelsey N. Thompson[1,2,3], Curtis Huttenhower[1,2,3,4], Eric A. Franzosa[1,2,3,*]

[1]Harvard Chan Microbiome in Public Health Center,
Harvard T. H. Chan School of Public Health, Boston, MA 02115, USA

[2]Department of Biostatistics,
Harvard T. H. Chan School of Public Health, Boston, MA 02115, USA

[3]Infectious Disease and Microbiome Program,
Broad Institute of MIT and Harvard, Cambridge, MA 02142, USA

[4]Department of Immunology and Infectious Diseases,
Harvard T. H. Chan School of Public Health, Boston, MA 02115, USA

*Corresponding author: Eric A. Franzosa

Supplementary Notes

Supplementary Note 1: Details of synthetic data generation

We simulated source microbial communities for metagenomes and metatranscriptomes using a SparseDOSSA-inspired model (https://huttenhower.sph.harvard.edu/sparsedossa) trained on the 100 most-prevalent species from MetaPhlAn 2 (Truong*, et al.*, 2015) profiles of the healthy human gut microbiome (Lloyd-Price*, et al.*, 2017). Each species was characterized by 1) a probability of being present (non-zero) equal to its empirical prevalence and 2) a log-normal abundance distribution fit to its non-zero values and truncated at three standard deviations from the mean. We simulated species biomass within a sample by first randomly determining if each of these 100 species was present and then drawing abundances for the present species from their individual log-normal distributions.

We modeled species pangenomes as pools of 1,000 molecular functions ("genes") drawn from a total pool of 2,000 such functions ("gene families" or "orthogroups"). Thus, the 2,000 gene families vary randomly with respect to their taxonomic distribution across the simulated species. When a species was present in a sample, we simulated a sample-specific strain carriage (i.e. differential carriage of genes compared to the broader species definition) by assuming that any of the species' 1,000 genes could be present with probability of 80% (the "encoding" frequency). The per-sample abundance of a gene was defined as the per-sample abundance of its encoding species if present and zero otherwise, thus modeling constant expected coverage across the species' genome. To simplify our models, we did not consider 1) variation in gene length, 2) variation in gene encoding frequency within species, nor 3) relationships between encoding frequency and taxonomic distribution.

Finally, to simulate gene expression, each gene family was assigned a (log) mean expression value (m) drawn from $N(0, 1)$. Sample-specific expression values for a given gene were then drawn from $\log N(m, 1)$ and truncated to 3 standard deviations from the mean. The per-sample transcriptional abundance of a gene was then defined as the product of its sample-specific expression value and the per-sample gene abundance.

The above process was used to generate synthetic datasets with N=100 samples. The samples were randomly divided into binary case and control groups (the sample "phenotype"). In order to induce associations between sample phenotype and species, gene encoding, and gene expression measurements, we semi-randomly redistributed features across samples such that ranked feature values fell within a $1 - s$ percentile window of the corresponding ranked metadata (where s was the strength of the induced association). For example, to induce a positive association with strength $s = 0.8$ between a species X and sample phenotype, the sample at the 60th percentile of the metadata would be paired randomly with a species X value in the 50-70th percentile of X values. Features associated to phenotype with strength s=1.0 are thus identically ranked (corresponding to a Spearman correlation of 1.0) before downstream normalization.

When reordering gene-encoding measurements, only samples where the source species was present were considered (i.e. the spiking procedure could not cause a gene to become present

when its source species was absent). Similarly, when reordering gene expression values, only samples where the corresponding gene was encoded were considered. Potentially confounding associations were always induced at maximum strength (*s* = 1.0), while associations with gene expression phenotypes were induced at *s* = 0.5 (low), 0.75 (medium), and 1.0 (high) strength in separate experiments. Half of all spiked associations were induced with a negative sign by initially reversing the ranks of feature measurements. Unless otherwise specified, when a given feature type was spiked with associations, 10% of features were selected at random. In some experiments, spike-in effects for gene encoding or expression were first determined at the gene-family level and then propagated to all "orthologs" of those genes across species. This enabled the creation of DE signals at the community level: e.g. all species encoding function *Y* strongly over-express the function in cases.

To simulate metagenomic sequencing, each sample was assigned a random read depth sampled uniformly from 10 to 20 million reads. Species and gene counts were then sampled from a multinomial distribution parameterized on the sample read depth and sum-normalized abundance values. In some experiments, we induced a maximum-strength (positive) association between sample read depth and phenotype prior to sampling feature reads. Procedures for simulating metatranscriptome sequencing (and resultant transcript counts) were identical to those described for metagenomes with one exception: the range of possible read depths was reduced to 5 to 10 million to account for the larger fraction of RNA reads derived from non-coding sequences in real-world community RNA sequencing.

Lloyd-Price, J., *et al.* (2017) Strains, functions and dynamics in the expanded Human Microbiome Project. *Nature,* **550**, 61-66.
Truong, D.T., *et al.* (2015) MetaPhlAn2 for enhanced metagenomic taxonomic profiling. *Nat Methods,* **12**, 902-903.

Supplementary Tables

**Synthetic dataset number: name (*main-text abbreviation*)**	**Detailed description of the dataset**
D1: No associations (*null*)	No meta-omic features are purposefully associated with sample case:control phenotype; hence, all genes are negative for differential expression (DE).
D2: Taxon confounding (*null-bug*)	The genomic (DNA) abundances of 10% of taxa are strongly associated with case:control phenotype, which may induce spurious signals of DE.
D3: Gene confounding (*null-enc*)	Gene presence/absence (encoding) of 10% of genes within taxa is strongly associated with case:control phenotype, which may induce spurious signals of DE.
D4: Read-depth confounding (*null-dep*)	Sequencing depth is strongly associated with sample phenotype (cases have higher average DNA and RNA read depth).
D5: Strong expression trends (*true-exp*)	The expression of 10% of community genes is strongly associated with sample phenotype (DE positives); all other genes are DE negatives.
D6: Moderate expression trends (*true-exp-med*)	The expression of 10% of community genes is moderately associated with sample phenotype.
D7: Weak expression trends (*true-exp-low*)	The expression of 10% of community genes is weakly associated with sample phenotype.
D8: Taxon + expression trends (*true-combo-bug-exp*)	50% of taxon abundances and the expression of 10% of community genes are strongly associated with sample phenotype (species and their transcripts can trend in opposite directions).
D9: Read-depth + expression trends (*true-combo-dep-exp*)	Sequencing depth is associated with sample phenotype, as is the expression of 10% of community genes.
D10: Community gene confounding (*group-null-enc*)	Gene presence/absence for 10% of gene families (spanning taxa) is strongly associated with sample phenotype. That is, if a given gene is lost in cases in one taxon, its orthologs are lost in cases in other taxa (but transcript levels are not associated).
D11: Community expression trends (*group-true-exp*)	Gene expression for 10% of gene families (spanning taxa) is strongly associated with sample labels. That is, if a given gene is up-regulated in cases in one taxon, its orthologs are up-regulated in cases in other taxa.

Supplementary Table 1: Detailed descriptions of synthetic datasets. Datasets are referring to by abbreviation "e.g. bull-bug" in the main text and all figures.

Choice 1: Method of gene-copy normalization	Choice 2: Method of zero-filtration	Choice 3: Link function	Choice 4: Sequencing depth covariate
M1: RNA normalized within-community	N/A	Linear / Logistic	yes / no
M2: RNA normalized within taxon	N/A	Linear / Logistic	yes / no
M3: RNA normalized within taxon with taxon total-RNA covariate	Lenient / semi-strict / strict	Linear / Logistic	yes / no
M4: RNA normalized by paired DNA using RNA/DNA ratio	Lenient / semi-strict / strict	Linear / Logistic	yes / no
M5: RNA normalized by paired total-taxon DNA covariate	Lenient / semi-strict / strict	Linear / Logistic	yes / no
M6: RNA normalized by paired DNA covariate	Lenient / semi-strict / strict	Linear / Logistic	yes / no

Supplementary Table 2: Statistical approaches for DE analysis in MTX data. We considered a large number of potential models for DE in MTX, each incorporating choices of 1) one of six gene-copy normalization methods, 2) optional pre-filtering of zero values by one of three strategies, 3) linear regression against transcript abundance vs. logistic regression against transcript presence/absence, and 4) the optional addition of a MTX read depth covariate within the model formulation. The performance of subsets of these model configurations are discussed in the main text, with a focus on the six normalization methods (M1-6).

Supplementary Figures

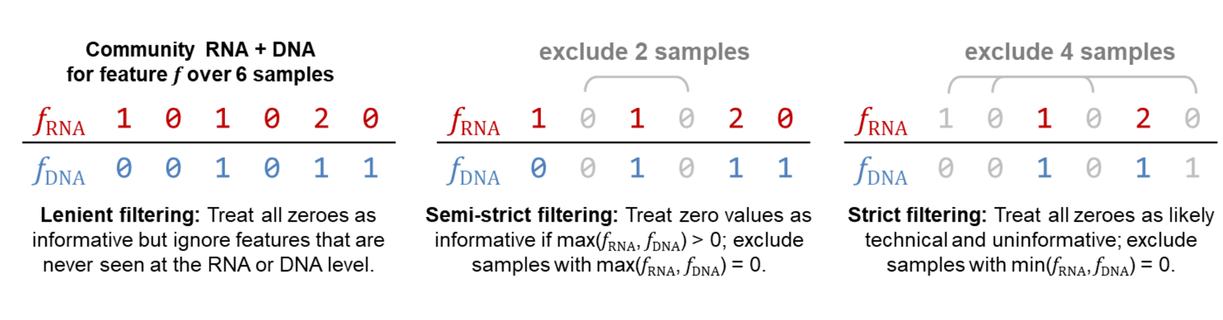

Supplementary Figure 1: Three strategies for pre-filtering potential technical zeroes from MTX data. Feature-level DNA abundance (f_{DNA}) is replaced by taxon-level estimates of this quantity under normalization models M3 and M5 and ignored by normalization models M1 and M2.

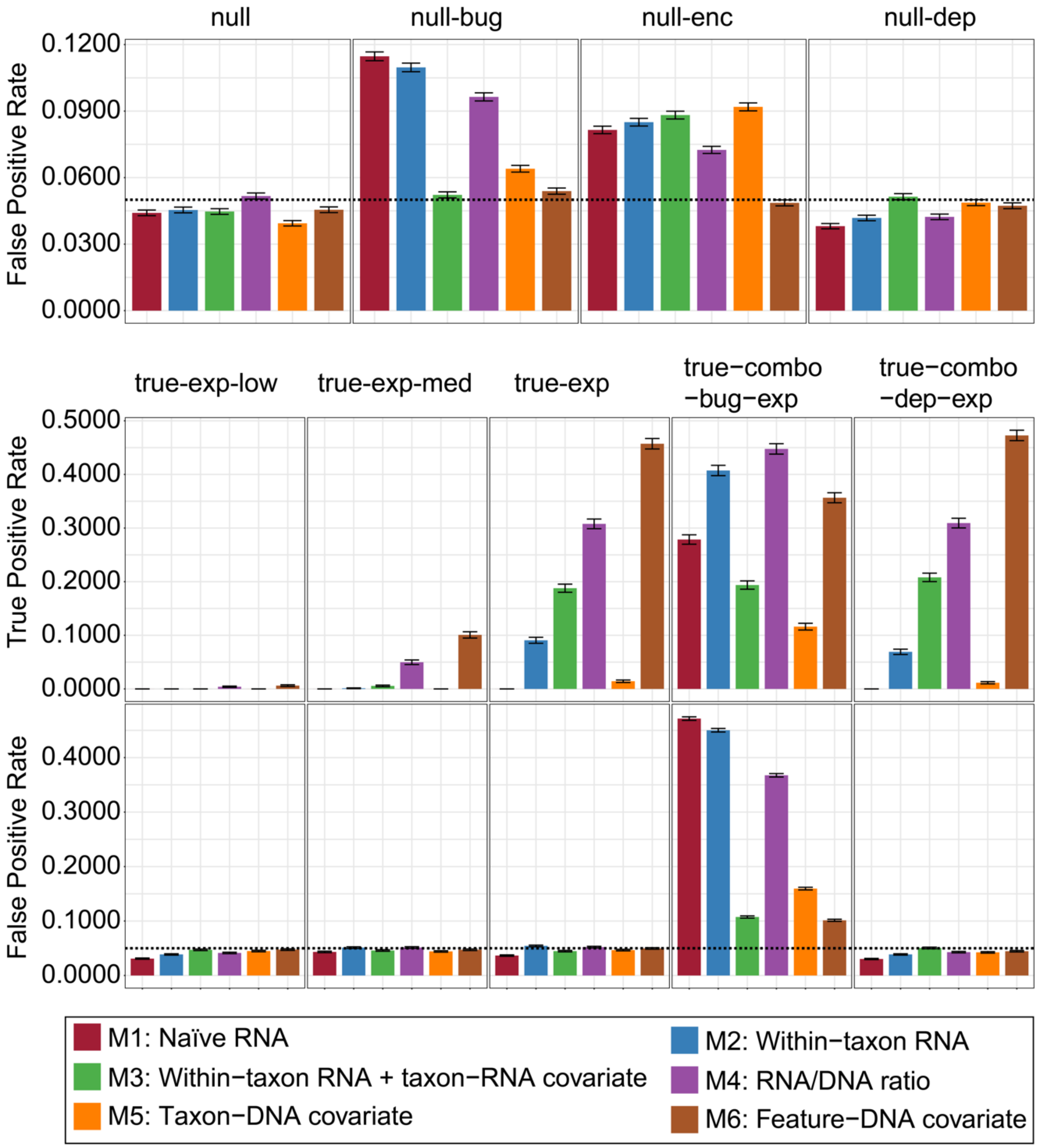

Supplementary Figure 2: Performance of six models for community DE on synthetic MTX and MGX datasets subjected to "lenient" pre-filtering of zero values. This figure provides a more detailed view of the performance measures from **Fig. 3A** of the main text; it is an analog of main-text **Fig. 2** incorporating "lenient" rather than "strict" filtering (see **Supplementary Fig. 1** for definitions). Error bars reflect the 95% CI for percentages.

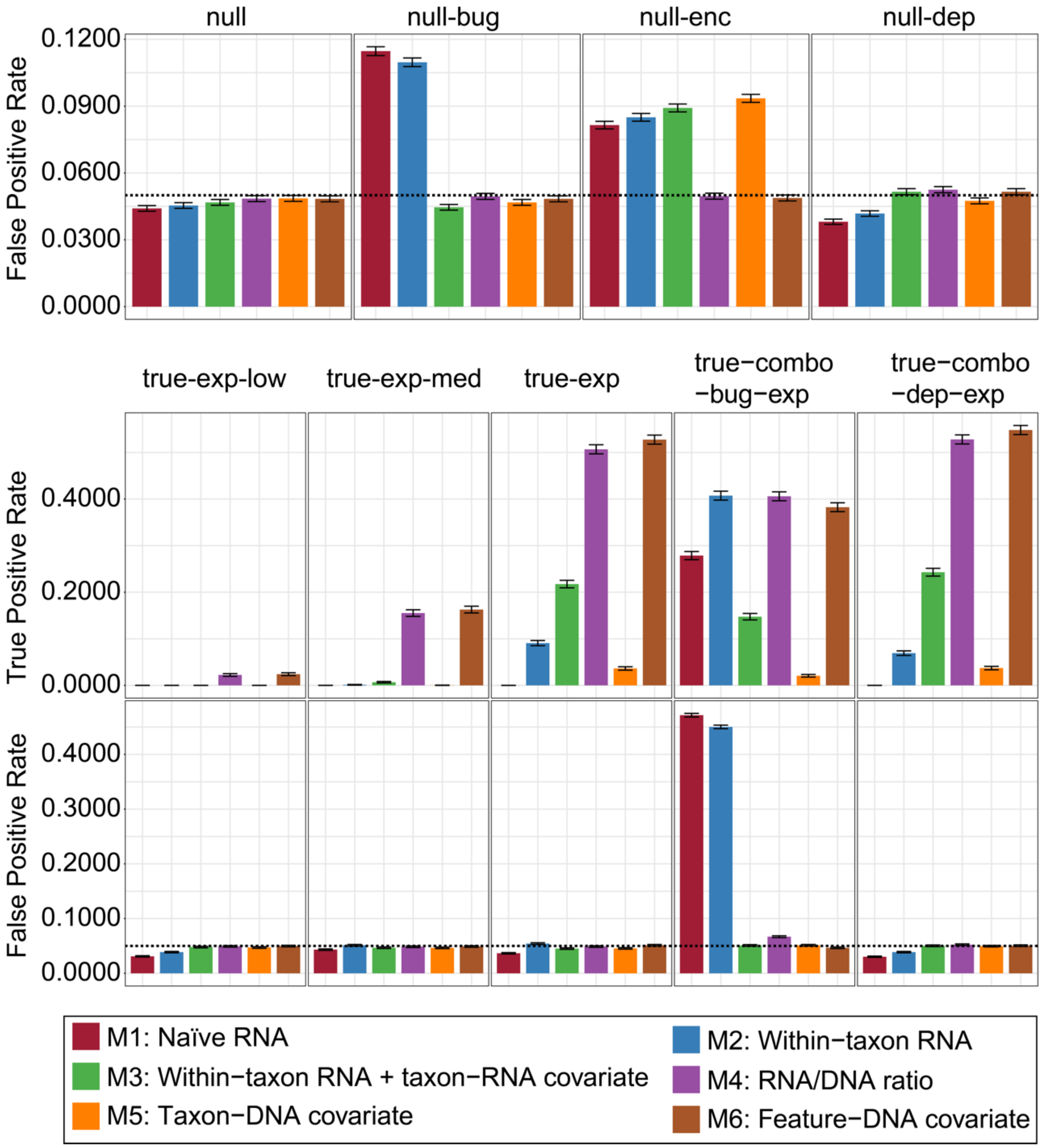

Supplementary Figure 3: Performance of six models for community DE on synthetic MTX and MGX datasets subjected to "semi-strict" pre-filtering of zero values. This figure provides a more detailed view of the performance measures from **Fig. 3B** of the main text; it is an analog of main-text **Fig. 2** incorporating "semi-strict" rather than "strict" filtering (see **Supplementary Fig. 1** for definitions). Error bars reflect the 95% CI for percentages.

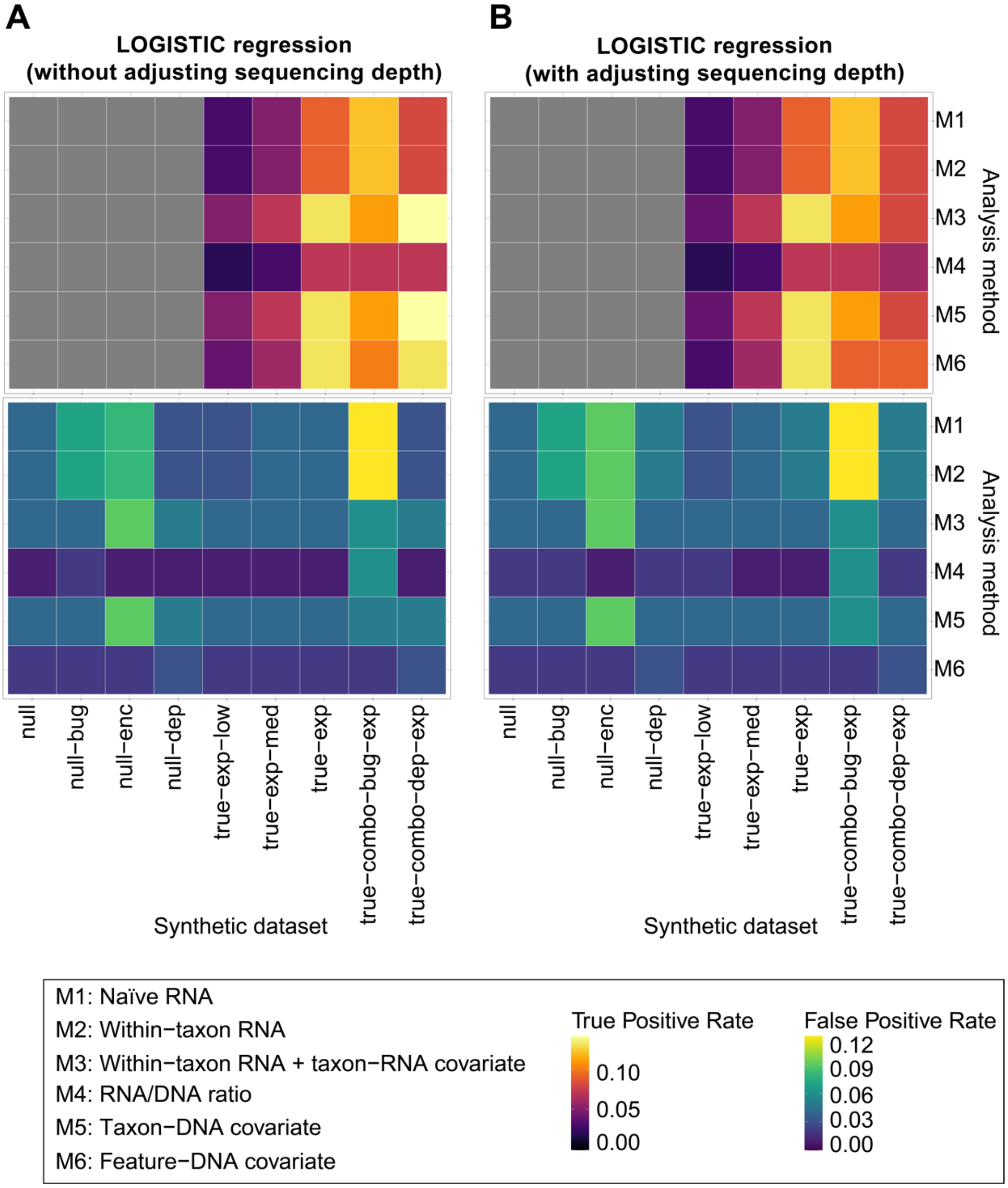

Supplementary Figure 4: Logistic models for community DE discovery from MTX data. This figure is an analog of **Fig. 3** from the main text based on associating transcript presence/absence with sample covariates via logistic regression (rather than associating transcript abundance with covariates via linear regression). (**A**) Logistic models evaluated without the addition of a separate MTX read depth covariate. (**B**) Evaluations incorporating a read depth covariate. Trends in presence/absence modeling were generally similar to abundance modeling but lower-powered. The addition of a read depth covariate did not improve performance.

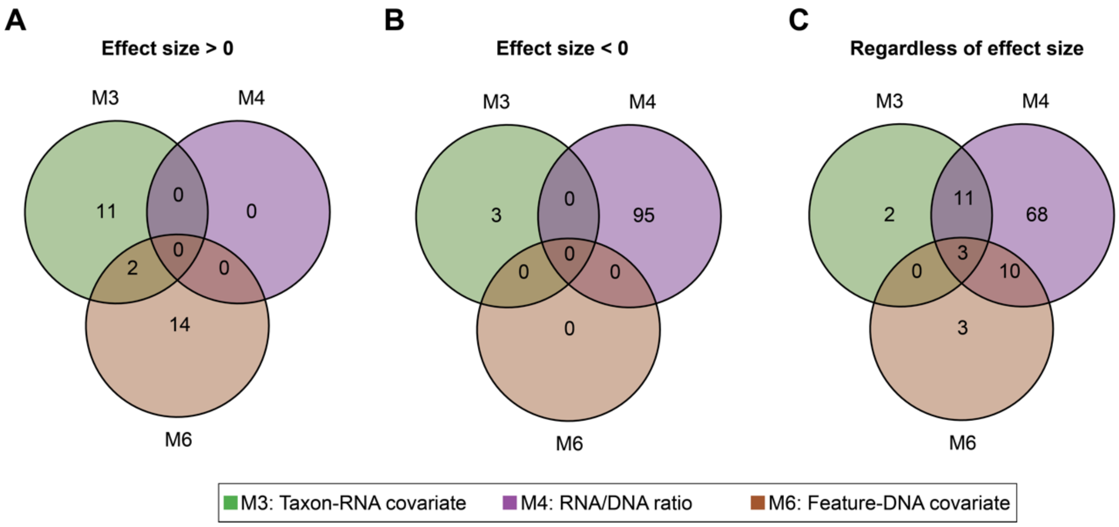

Supplementary Figure 5: Comparison of *E. coli* pilin-like proteins that were considered DE in MTX of the IBD gut microbiome under three models of community DE. Venn diagrams compare overlaps in sets of proteins with FDR significant DE (FDR $q < 0.25$) under the taxon-RNA covariate model (M3), RNA/DNA ratio model (M4), and feature-DNA covariate model (M6) focusing on (**A**) positive effect sizes (i.e. increased pilin expression in dysbiosis), (**B**) negative effect sizes (i.e. decreased pilin expression in dysbiosis), or (**C**) their union. Based on the semi-strict filtration of zeros from this analysis, we hypothesize that a subset of M3-specific associations results from an inability to differentiate down-regulation from gene absence using M3's taxon-level estimate of gene abundance; similarly, the preponderance of down-regulation trends reported by M4 may be driven by excessive depression of the RNA/DNA ratio by dysbiosis-enriched *E. coli* genomes.
